# The Applications of Metaheuristics for Human Activity Recognition and Fall Detection Using Wearable Sensors: A Comprehensive Analysis

**DOI:** 10.3390/bios12100821

**Published:** 2022-10-03

**Authors:** Mohammed A. A. Al-qaness, Ahmed M. Helmi, Abdelghani Dahou, Mohamed Abd Elaziz

**Affiliations:** 1 College of Physics and Electronic Information Engineering, Zhejiang Normal University, Jinhua 321004, China; 2 College of Engineering and Information Technology, Buraydah Private Colleges, Buraydah 51418, Saudi Arabia; 3 Computer and Systems Engineering Department, Faculty of Engineering, Zagazig University, Zagazig 44519, Egypt; 4 Mathematics and Computer Science Department, University of Ahmed DRAIA, Adrar 01000, Algeria; 5 LDDI Laboratory, Faculty of Science and Technology, University of Ahmed DRAIA, Adrar 01000, Algeria; 6 Department of Mathematics, Faculty of Science, Zagazig University, Zagazig 44519, Egypt; 7 Artificial Intelligence Research Center (AIRC), College of Engineering and Information Technology, Ajman University, Ajman 346, United Arab Emirates; 8 Faculty of Computer Science and Engineering, Galala University, Suez 435611, Egypt; 9 Department of Electrical and Computer Engineering, Lebanese American University, Byblos 13-5053, Lebanon

**Keywords:** fall detection, human activity recognition wearable sensors, deep learning (DL), convolution neural network

## Abstract

In this paper, we study the applications of metaheuristics (MH) optimization algorithms in human activity recognition (HAR) and fall detection based on sensor data. It is known that MH algorithms have been utilized in complex engineering and optimization problems, including feature selection (FS). Thus, in this regard, this paper used nine MH algorithms as FS methods to boost the classification accuracy of the HAR and fall detection applications. The applied MH were the Aquila optimizer (AO), arithmetic optimization algorithm (AOA), marine predators algorithm (MPA), artificial bee colony (ABC) algorithm, genetic algorithm (GA), slime mold algorithm (SMA), grey wolf optimizer (GWO), whale optimization algorithm (WOA), and particle swarm optimization algorithm (PSO). First, we applied efficient prepossessing and segmentation methods to reveal the motion patterns and reduce the time complexities. Second, we developed a light feature extraction technique using advanced deep learning approaches. The developed model was ResRNN and was composed of several building blocks from deep learning networks including convolution neural networks (CNN), residual networks, and bidirectional recurrent neural networks (BiRNN). Third, we applied the mentioned MH algorithms to select the optimal features and boost classification accuracy. Finally, the support vector machine and random forest classifiers were employed to classify each activity in the case of multi-classification and to detect fall and non-fall actions in the case of binary classification. We used seven different and complex datasets for the multi-classification case: the PAMMP2, Sis-Fall, UniMiB SHAR, OPPORTUNITY, WISDM, UCI-HAR, and KU-HAR datasets. In addition, we used the Sis-Fall dataset for the binary classification (fall detection). We compared the results of the nine MH optimization methods using different performance indicators. We concluded that MH optimization algorithms had promising performance in HAR and fall detection applications.

## 1. Introduction

### 1.1. Motivation

Many context-awareness services allow computers to track and recognize human motion and activities. It is clear that smart environments can be investigated due to the increased usage of smart devices in homes as well as Internet of things (IoT)-supported devices [1]. Human activity recognition (HAR) can be defined as the process of identifying a person’s actions from a series of measurements captured by different mechanisms such as cameras (computer vision mechanism) [2], interior sensors [3,4], radars [5], wireless signals [6], and others.

This study focuses only on the HAR applications that use sensor data. Sensor-based HAR mechanisms have the advantage of being able to collect sensing data at any time and in any location as well as obtain information that is unique to each user. The disadvantage is that each user must own the sensor equipment; however, the widespread popularity of smartphones and smartwatches has solved this problem. In addition, there are still some issues, such as different measurement conditions, e.g., device kind; the installed position of the sensors; the wearing technique; and the measuring applications, which differ from one user to the next and from one measurement date to the next. To solve these challenges, in this study, we use different and complex datasets that cover all types of sensor data proposed for HAR applications.

The classification process in HAR applications is a challenge due to the complexity of the sensors’ datasets. Thus, the feature selection process plays a significant role in HAR applications to reduce computation time and select only the optimal feature set. The selected MH optimization algorithms have been widely employed for feature selection in different domains. For example, the AO was utilized as an FS technique to enhance the intrusion detection system in the IoT and cloud environments [7,8]. In [9], the AO was employed as an FS for COVID-19 image classification. The AOA was utilized in various FS tasks such as osteosarcoma detection [10] and our previous study of HAR [11]. The MPA was also utilized in different applications, such as COVID-19 CT image classification [12], breast cancer classification [13], and wind power forecasting [14]. The SMA was adopted in different FS applications such as medical data classification [15], parameter identification of photovoltaic systems [16], and crude oil forecasting. The GA was also utilized for different applications such as lung cancer classification [17], data mining [18], and credit risk assessment [19]. The GWO was adopted in advanced applications such as mammogram image analysis [20], Parkinson’s disease diagnosis [21], and chronic disease prediction [22]. The WOA was applied in several FA applications such as email spam detection [23], software fault prediction [24], and medical data classification [25]. The ABC was adopted in different FS tasks such as colon cancer detection [26], medical image classification [27], and IDS [28]. The PSO is one of earlier methods that was employed for different FS such as data mining in the oil industry [29], breast cancer recurrence prediction [30], and intrusion detection system (IDS) [31].

### 1.2. Paper—Main Contributions

In this paper, we propose an efficient HAR approach that can be utilized for multi-classification (for different daily activities) and binary classification (for fall detection, including fall or non-fall action). The developed approach depends on two main processes. The first is feature extraction in which a light deep learning (DL) approach called ResRNN is developed to extract a subset of features that represent human motion from the sensor data. The ResRNN is built based on several building blocks from DL networks including convolution neural networks (CNN), residual networks, and bidirectional recurrent neural network (BiRNN). The second approach is to leverage recent advances in MH optimization algorithms in feature selection. We tested nine MH algorithms in feature selection to build an efficient HAR system, namely the Aquila optimizer (AO), arithmetic optimization algorithm (AOA), marine predators algorithm (MPA), artificial bee colony (ABC) algorithm, genetic algorithm (GA), slime mold algorithm (SMA), grey wolf optimizer (GWO), whale optimization algorithm (WOA), and particle swarm optimization algorithm (PSO). The main problems of sensor-based HAR approaches are the different positions of sensors in the human body, the types of motions, the complexities of human activities, the number of activities, and the number of tested users (the people who implemented the tested activities). To solve those challenges and comprehensively analyze the MH algorithms’ applications in HAR applications, we used seven datasets that covered all the mentioned challenges, namely, UCI-HAR, WISDM, UniMiB SHAR, OPPORTUNITY, KU-HAR, Sis-Fall, and PAMMP2. To sum up, we present the following contributions to the field of HAR:

- We studied the impacts of metaheuristic (MH) optimization algorithms on human activity recognition (HAR) and fall detection using body-attached sensor data. We tested nine MH algorithms and compared their performances.
- We developed a light feature extraction approach called ResRNN using several deep learning models, such as convolution neural networks (CNN), residual networks, and bidirectional recurrent neural network (BiRNN), to expose the related features from the collected signal data.
- We examined the suggested feature selection methods based on MH algorithms using different and complex datasets that covered all the aspects of sensor data for HAR and fall-detection applications.

### 1.3. Paper—Organization

In Section 2, we describe several recently published studies for sensor-based human activity recognition using different datasets. In Section 3, we describe the preliminaries of the applied methods including the nine MH optimization algorithms, the basic definitions of the seven datasets, the developed feature extraction method, and the feature optimization process. Moreover, the evaluation experiments’ settings, evaluation, comparison, and results are presented in Section 4. We conclude the paper in Section 6.

## 2. Related Work

In this section, we present a quick review of the previous studies proposed for senor-based HAR applications using different public datasets. Deep learning models have been widely utilized in different fields [32] including HAR and fall detection.

In [33], the authors proposed a new deep learning method using a hybrid gated recurrent unit (GRU) and LSTM -based RNN model for HAR. They used the TRECVID dataset to test the performance of the proposed deep learning model, which showed significant performance. Wang et al. [34] applied the traditional CNN model with an attention mechanism to extract sensor data features. They used the well-known UCI-HAR dataset to assess the performance of the CNN-based HAR model. Xia et al. [35] applied a hybrid LSTM and CNN to classify different human activities using three public datasets: OPPORTUNITY, UCI-HAR, and WISDM. The main goal of this combined model was to automatically extract features from the sensor data with fewer parameters to reduce the computation complexity. Sikder et al. [36] employed a sequential deep learning method to classify human activities using three public datasets: UCI-HAR, KU-HAR, and WISDM. They applied a specific preprocessing method using a specific matrix formulated from sensor data. The classification outcomes achieved high accuracy rates for the three datasets. Kumar and Suresh [37] proposed a new HAR model called DeepTransHHAR using deep learning with heterogeneous deep transfer learning. They used two well-known datasets, KU-HAR and HHAR, to evaluate the developed DeepTransHHAR, achieving acceptable classification results. Dua et al. [38] presented a combined deep learning model using the CNN and GUR to extract features automatically and recognize implemented activities. They evaluated the combined CNN-GRU with three public datasets, PAMAP2, WISDM, and UCI-HAR. Khatun et al. [39] proposed a combined deep learning model (CNN-LSTM) with a self-attention mechanism for HAR smartphone applications. They assessed the quality of the combined model using two benchmark datasets, UCI-HAR and MHEALTH. Ghate et al. [40] applied a hybrid of deep CNN with the random forest classifier to classify human activities using two datasets, WISDM and UCI-HAR. It was compared to other models, such as LSTM, GRU, and CNN, and they found that CNN with RF obtained the best classification accuracy. Ronald et al. [41] developed a deep leering model based on Inception-ResNet architecture called iSPLInception to classify different human activities. They assessed the quality of the proposed model with four well-known HAR datasets, UCI HAR, OPPORTUNITY, PAMAP2, and Daphnet freezing of the gait dataset. Tufek et al. [42] studied the capability of the LSTM and CNN models to classify human activities based on sensor data. They used the UCI-HAR dataset to evaluate both the LSTM and CNN models. They found that the LSTM model had better classification accuracy than the CNN model. Gao et al. [43] developed a new HAR model based on a multi-branch CNN architecture using a selective-kernel mechanism. They employed four datasets to test the performance of the developed CNN model, namely UCI-HAR, WISDM, UniMiB SHAR, OPPORTUNITY, and PAMAP2. Huang et al. [44] employed a shallow CNN model with channel selectivity for HAR applications. They used five benchmark datasets to verify the performance of the CNN-based HAR model called OPPORTUNITY, namely the UCI-HAR, PAMAP2, WISDM, and UniMiB SHAR datasets. Gao et al. [45] suggested a dual attention deep learning approach, namely DanHAR, for HAR applications using sensor data. This model can blend channel and temporal attention on the residual network to enhance feature representation capability. The WISDM, UniMiB SHAR, PAMAP2, and OPPORTUNITY datasets were utilized to assess the classification capability of the developed DanHAR model, which obtained higher accuracy rates than several other models. Tang et al. [46] presented a CNN-based HAR method. The main idea was to boost the multi-scale feature representation capability using one feature layer. The UniMiB SHAR, PAMAP2, UCI-HAR, and WISDM datasets were utilized to assess the method performance with comparisons to several CNN models. The outcomes showed that the applied CNN-based feature extraction approach boosted the classification accuracy of all datasets.

It is worth mentioning that MH optimization algorithms including SI methods have limited applications in HAR systems. Almost all of the MH and SI method applications were in computer vision-based HAR applications such as the PSO [47,48] and genetic algorithm (GA) [49]. For sensor data, we carried out a simple implementation for the arithmetic optimization algorithm (AOA) with the KU-HAR, UCI-HAR, and WISDM datasets from [11] and the grey wolf optimizer using WISDM and UCI-HAR [50].

Unlike previous approaches, this paper presents a comprehensive analysis of MH optimization algorithms in HAR applications using sensor data. This is the first comprehensive study that investigates nine MH algorithms in HAR applications with seven public datasets, including comprehensive as well as normal and complex daily activities. The paper opens a new direction for further investigating MH optimization algorithms to boost HAR applications based on smart devices installed with embedded sensors.

## 3. Materials and Methods

**Figure 1:**
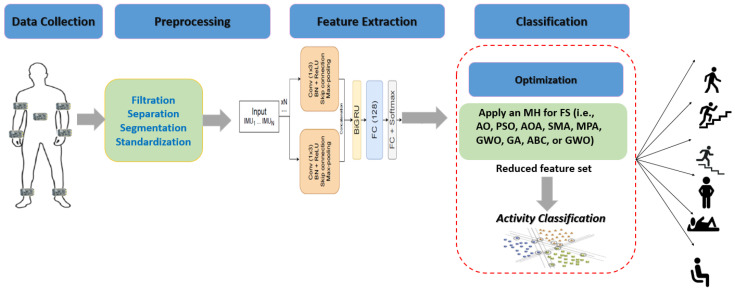
The main workflow of HAR application using the integration of deep learning and MH optimization algorithms.

In this section, we describe the preliminaries and the backgrounds of the used HAR datasets (KU-HAR, UCI-HAR, WISDM, PAMAP2, OPPORTUNITY, SiS-Fall, and UniMiB SHAR) and the applied feature selection methods based on different MH optimization algorithms, namely the Aquila optimizer (AO), arithmetic optimization algorithm (AOA), marine predators algorithm (MPA), artificial bee colony (ABC) algorithm, genetic algorithm (GA), slime mold algorithm (SMA), grey wolf optimizer (GWO), whale optimization algorithm (WOA), and particle swarm optimization algorithm (PSO). The main workflow of the HAR approach described in this paper can be seen in Figure 1. It consists of four main stages. The first is the data collection from the body-attached sensors. In this study, we used seven public datasets containing comprehensive and complex activities in different environments and were collected by different people in different countries. The second stage is data prepossessing. We used different cleaning and filtering methods to obtain clean data that can expose the impact of human activities on the collected signals. The third stage is the feature extraction stage. In this paper, we developed a new deep learning model to extract relevant features from the prepossessed datasets. The model is called ResRNN. It consists of several building blocks using different deep learning structures. (BiRNN). The fourth stage is the classification stage. In this stage, MH optimization methods are employed to select optimal features, reduce computation complexity, and optimize the classification process. Then, a classifier can be employed to recognize the implemented activities. This study tested and compared two well-known classifiers, the SVM and RF classification models.

### 3.1. Experimental HAR Datasets

#### 3.1.1. KU-HAR

**Figure 2:**
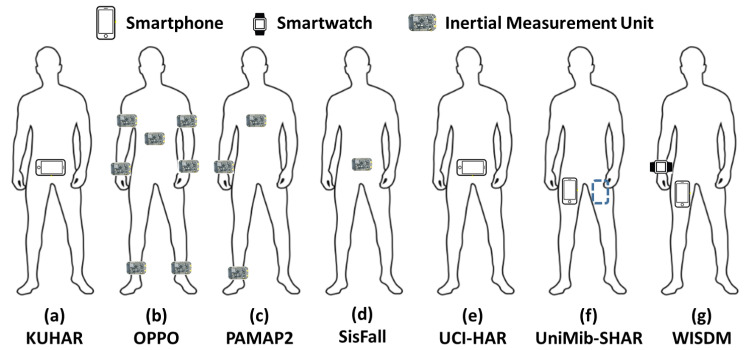
Sensor placement on the subject’s body. A waist-mounted smartphone was used for KUHAR, UCI-HAR, and WISDOM. WISDOM was collected using additional smartwatches. IMU units were used to collect the signals for OPPO (positions: back, right/left upper/lateral parts of the arm and right/left shoe ), PAMAP2 (positions: chest, wrist, and ankle), and Sis-Fall (position: waist).

Skider and Nahid [51] collected and built a new dataset for human daily activities and actions, called the KU-HAR dataset. They used the gyroscopes and accelerometers of smartphones (See Figure 2a). They collected 1945 raw samples for 18 different classes (human activities) that were implemented by 90 users aged between 18 and 34. The collected samples contained 20,750 subsamples, each with a period of 3 s.

#### 3.1.2. OPPORTUNITY (Oppo)

The OPPORTUNITY dataset was collected by [52] using wearable inertia measurement units (IMUs) that were placed on human users’ bodies at seven different positions (see Figure 2b). Four human users implemented 18 daily motions and activities in a simulation room, such as “close/open doors/fridges/drawers, clear tables, toggle switches, and sip from cups,” and others. The collected samples had 77-dimensional attribute columns that characterized the activity signals and have been sampled at a frequency of 30 Hz. It is worth mentioning that the “emphNULL” class represents 72% of all collected samples, which represents a non-relevant activity. More details can be found in [52].

#### 3.1.3. PAMAP2

The PAMAP2 dataset was collected by [53] with nine human users who implemented different daily activities. The users were asked to wear three IMUs at the ankle, chest, and wrist positions, as clarified in Figure 2c. During data collection, for each activity (one motion), the magnetometer, gyroscope, and accelerometer reported 27-dimensional signal spaces at a frequency rate of 100 Hz. A total of 2,844,868 raw samples for 12 different human activities and motions were collected during the data collection period.

#### 3.1.4. Sis-Fall

The SiS-Fall dataset was collected by [54] using an embedded unit with a Kinets MKL25Z128VLK4 microcontroller, which had two accelerometers (MMA8451Q and ADXL345) and one ITG3200 gyroscope (see Figure 2d). It contained two types of human motions; the first was called activities of daily living (ADLs) and the second was called fall actions. The ADL type contained 19 activities: jogging, walking, walking downstairs and upstairs, standing, jumping, sitting, stumbling while walking, and different sitting motions. The fall type contained 15 fall actions such as fall forward, fall backward, lateral fall, fall while jogging, fall while getting up, fall while sitting down, and others. A total of 38 human users implemented the activities, with 15 elderly users (7 female and 8 male) and 23 young people (12 female and 11 male).

#### 3.1.5. UCI-HAR

In the collection stage of the UCI-HAR dataset, a Samsung Galaxy SII smartphone was used [55] (see Figure 2e). A total of 30 users implemented six daily activities: walking, walking upstairs, walking downstairs, sitting, standing, and lying. The gyroscope and accelerometer records were collected separately. Thus, a total of 10,299 samples were collected at a rate of 50 Hz, with a sliding window of 2.56 s.

#### 3.1.6. UniMiB SHAR

In [56], a new HAR dataset was collected based on the accelerometers of smartphones. During the data collection stage, the users were asked to put the smartphone in their pockets (right and left), as shown in Figure 2f. The UniMiB SHAR had two types of human motions: daily activities and fall actions. The first contained nine activities, (“Running, Walking, Standing UPFS (Standing up from sitting), Jumping, Standing UPFL (Standing up from lying), Going Ups (Going upstairs), Going Downs (Going downstairs), sitting down, and Lying DownFS (Lying down from standing)”). The second type had eight fall actions (“Hitting Obstacle, Falling right, Falling Back, Falling Left, Falling BackSC (Falling backward sitting chair), Falling with protection strategy (Falling withPS), Falling Forward, and Syncope”). A total of 30 human users were asked to implement the mentioned motions, and 11,771 raw samples were collected during the experiments.

#### 3.1.7. WISDM

The wireless sensor data mining (WISDM) dataset was collected by [57] using Android smart devices (i.e., smartwatches and smartphones; see Figure 2g) ). A total of 36 users implemented six physical activities, such as jogging, walking, sitting, upstairs, downstairs, and standing. A total of 1,098,207 raw samples were collected during the experiments, where each sample indicated an accelerometer data measurement (at 20 Hz).

### 3.2. Applied Metaheuristic Optimization Algorithms

#### 3.2.1. Aquila Optimizer (AO)

The AO algorithm was developed by [58]. It is a population-based MH optimization method. The main idea of the AO is to mimic aquilas’ natural behavior in catching prey in the wild. The workflow and detailed description of the AO can be found in [58].

#### 3.2.2. Arithmetic Optimization Algorithm (AOA)

The AOA optimization method was developed by [59] and was inspired by basic mathematical operations (i.e., −,+,*, and /). The basic steps and detailed description of the workflow of the AOA can be found in [59].

#### 3.2.3. Marine Predators Algorithm (MPA)

The MPA was developed by [60] and was inspired by the natural behavior of predators and prey. The prey and predators can be considered search agents, whereas a predator searches for prey and a prey searches for food. Similar to other MH techniques, it starts by generating a set of solutions (agents) as an initialization. After that, the agents can be modified depending on the main workflow of the algorithm. More details and mathematical descriptions can be found in [60].

#### 3.2.4. Slime Mold Algorithm

The SMA was developed by [61] as a natural-inspired MH technique and belongs to swarm intelligence-inspired algorithms. The main idea of the SMA is to mimic the natural behaviors of slime mold oscillations and their propagation wave feedback depending on the bio-oscillator. It generates the optimum routes to connect food. More details can be found in [61].

#### 3.2.5. Whale Optimization Algorithm (WOA)

The WOA was proposed by [62] as a natural swarm intelligence method. It was inspired by the behavior of humpback whales in nature. In contrast, the whale’s position indicates the agent’s solution to a problem and it can be updated depending on the whale’s behavior when attacking prey. Two attacking techniques are used in the mathematical definition of the WOA. More details can be found in [62].

#### 3.2.6. Artificial Bee Colony (ABC) Algorithm

The ABC algorithm is a swarm intelligence-based MH method developed by Karaboga in 2005 [63] to solve complex optimization, numerical, and engineering problems. The ABC was inspired by the intelligent foraging behavior of honey bees. The ABC generally depends on the model developed by [64] based on the foraging behavior of honey bee colonies. This model has three phases, employed and unemployed foraging bees and food sources. The employed and unemployed bees are searching for food sources. This behavior can be represented in the ABC algorithm by generating a population that has agents. Thus, an agent (a colony of artificial forager bees) searches for a good solution (a good food source) (good solutions for a given problem). More details about the ABC algorithm can be found in [65]

#### 3.2.7. Grey Wolf Optimizer (GWO)

The GWO is a swarm intelligence-based method inspired by the natural behavior of Canis lupus (grey wolves). It was developed by [66] and received wide attention for solving different optimization problems. It simulates the leadership hierarchy’s natural behavior and grey wolves’ hunting processes. Four kinds of grey wolves (including alpha, beta, omega, and delta) are applied to simulate the leadership hierarchy. Additionally, there are three main steps in the hunting process: searching for prey, encircling prey, and attacking prey. The mathematical definition and a more detailed description can be found in [66].

#### 3.2.8. Genetic Algorithm

The GA was presented by [67] as a population-based MH algorithm. It can be considered an evolution-based algorithm. In the definition of the GA, each individual (agent) in the population is referred to as the solution. It has three phases that can be utilized for updating the solutions (agents) called the selection, crossover, and mutation processes. In the first phase, two agents can be randomly selected to boost the diversity of the population. The second phase, the crossover mechanism, can generate new agents (individuals) from the selected agents (parents). After that, the mutation process can be utilized to replace randomly selected agents with random values belonging to the search domain. Lastly, the current population can be updated based on the fitness values of the newly initialized agents and their parents. Furthermore, the population can be updated using the three processes mentioned (selection, crossover, and mutation) until the stop criteria are met. The mathematical definition and a detailed description of the GA can be found in [68].

#### 3.2.9. Particle Swarm Optimization (PSO)

The PSO is a population-based intelligence method proposed by [69]. It is one of the earliest swarm intelligence methods and has received much attention in previous decades. The PSO’s main workflow and mathematical details can be found in [69].

### 3.3. Data Cleaning, Filtration, and Segmentation

In general, HAR applications are considered real-time applications in which the recognition process must be performed immediately. Thus, the time window is generally set between 2 and 10 s [70,71,72]. In this section, we briefly describe the data preparation for all seven employed datasets.

**KU-HAR.** The KU-HAR dataset samples contain 300 data points; each segment is 3 s with non-overlapping, as described in the original study of the KU-HAR dataset [51]. At the same time, only one accelerometer was used to collect the tracing human activity samples. The Butterworth lowpass filter was applied to remove noise and filter the collected signals.

**OPPORTUNITY (Oppo).** In the Oppo dataset, there are many NAN values. The collected samples were gathered using wearable IMUs at a low-frequency of 30 Hz. Thus, a light linear interpolation was performed to prepare the collected samples. Following [45,73], the time window was set to 2.133 s, which had 64 data points. Furthermore, overlapping was employed for the segmentation process at a rate of 50%.

**PAMAP2.** To process the PAMAP2 collected samples, a lowpass filter called the Butterworth filter was used with a cut-off frequency of 20 Hz. The time window for activity segmentation was set to 5 s [45,74]. Overlapping also was used at a rate of 50% for data segmentation.

**SisFall.** Following previous studies such as [75], for this dataset, the time window was set to 3 s, in which 600 points were collected for each activity sample. A lowpass filter was also utilized to remove noise, as suggested by the original study of the Sis-Fall dataset [54].

**UCI-HAR.** The samples of the UCI-HAR dataset were segmented with a time window of 2.56 s, with a frequency of 50 Hz. Also, overlapping with 50% was performed. More details can be found in the original study [55].

**UniMiB SHAR.** The segmentation of the UniMiB SHAR dataset was set by the original study [56]. A time window of 3 s was used, with 150 data points for each activity sample.

**WISDM.** Similar to the other datasets, the Butterworth lowpass filter was employed to remove the noise from the collected samples, with a cut–off frequency of 10 Hz. For the collected samples, a time window with a length of 128 s was applied. Also, overlapping with 50% was implemented to train the model, as carried out by [71,76].

It is worth mentioning that we performed standardization on the raw data before feeding it to the DL model, where the input signals were scaled to unit variance along with removing the mean as in Equation (1).

(1) $$z=\frac{\left(x-u\right)}{s}$$

Where the mean and standard deviation of sample data, *x*, are represented by *u* and *s*, respectively. And thus *z* becomes the corresponding standard score.

### 3.4. Feature Extraction

**Figure 3:**
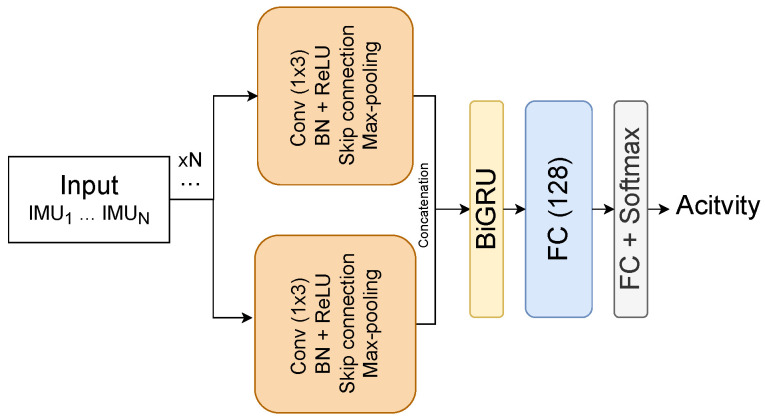
The proposed ResRNN model for feature extraction.

In this section, we describe the DL model used to extract features from preprocessed input data to learn better representations of the raw input data. The proposed DL model named ResRNN was composed of several building blocks from various DL networks including convolution neural networks (CNN), residual networks, and bidirectional recurrent neural networks (BiRNN). The ResRNN used parallel and sequential alignment of the components based on their structures. For instance, the CNN layers aligned parallel to extract different feature maps from the input data and learn activity nature-related features. The BiRNN layers were aligned in sequence to learn more complex and temporal-related features based on the previous layer’s output (CNN output). The input data were composed of triaxial signals (X, Y, Z), which represented each data sample based on the collected IMU signals from sensors such as the accelerometer, gyroscope, and magnetometer. At the beginning of the ResRNN, a set of convolution blocks aligned in parallel received the input data and extracted the feature maps. Based on the IMU signals collected from each sensor, the signals were distributed over the parallel convolution block with the following structure: (Conv→BN→ReLU→RC→Max−pooling). The Conv represented a convolution layer with a kernel size of (1×3), a stride of 1, padding of 1, and several filters of 64, 128, and 256 for the triaxial signals. The BN layer was a batch normalization layer. The ReLU layer was a Rectified Linear Unit activation layer. The RC layer was a residual connection similar to the skip connection mechanism in residual networks. The max-pooling layer was a max-pooling layer with a kernel size of 2 and a stride of 2. The learned feature maps from all parallel CNN blocks with different output channels were concatenated and fed to a BiRNN block with gated recurrent units (GRU) as the RNN structure and attention mechanism. The ResRNN benefited from the DL techniques as it could learn more complex representations and lower the computation complexity, thus generating a small model size, overcoming over-fitting and reducing the training time. The learned features from the BiGRU with the attention block were concatenated and fed to a fully connected layer (FC) with 128 neurons, which served as our feature extraction layer. Thus, the fine-tuned features in the FC layer were extracted and inputted into the feature selection algorithm. Each input sample was represented by a feature vector of size 128 extracted from the FC layer. The ResRNN’s weight was optimized for the activity classification task using the Adam optimizer with a 1 × 10${}^{-4}$ learning rate. A softmax layer was placed at the top of the model to perform the classification. In addition, the ResRNN was trained for 350 epochs with dropout (0.3) and early stopping set to monitor the validation loss. Figure 3 shows the architecture of the ResRNN.

### 3.5. Feature Optimization

Let *F* represent the feature vector extracted by the proposed DL model, then Equation (2) can be applied to characterize the model performance using the optimized feature vector S according to both the classification rate improvement and feature set reduction.

(2) $$↓f\left(S\right)=\alpha {CE}_{S}+\left(1-\alpha \right)\left(\frac{\left|S\right|}{\left|F\right|}\right),$$

where $CE_{S}$ represents model *classification error* for *S*, α∈[0,1] is a commonly applied balance factor to control the effect of $CE_{S}$ and the feature set optimization $\left(\frac{\left|S\right|}{\left|F\right|}\right)$, where |F| denotes the feature set cardinality. Thus, a lower value, i.e., f→0, in Equation (2) refers to the higher performance of the working model and vise-versa. In this work, $CE_{S}$ is set as the model classification accuracy defined by Equation (3).

(3) $$Accuracy\left(Acc\right)=\frac{TP}{TP+TN+FP+FN}$$

where TP, TN, FP, and FN are the model rates of true positive, true negative, false positive, and false negative, respectively. Algorithm 1 illustrates the designed cost function to work under the applied MH algorithms here, where α is set to 0.99 and thr=0.
**Algorithm 1***Cost Function* (S, $T_{tr}$, $T_{tst}$, α, thr)1:**Input:** Solution S, Train $T_{tr}$ and test $T_{tst}$ sets, control parameter α and threshold thr.2:**Output:** Solution cost (*f*)3:f←∞4:Obtain the equivalent binary solution $S^{B}\leftarrow S>thr$5:Select the features indicated by ones in $S^{B}$ in each of $T_{tr}$ and $T_{tst}$6:Train the classification layer in the model using the selected features in $T_{tr}$7:Calculate the classification predictions of the trained model for $T_{tst}$8:Calculate *f* using Equation (2)

## 4. Experiments

### 4.1. Experiments Setup

Table 1 presents the parameter settings of each MH algorithm applied in this work. For efficient computational overheads, the search settings were set to a population size of 20 and a total number of iterations of 20. To characterize the average performance of each applied MH algorithm, each experiment was repeated for 20 independent runs. Both binary and multi-class classification tasks were carried out using the SVM algorithm [77] and RF algorithm [78] under the ’Bag’ learning method, where the size of decision trees was set to 50.

In addition to *Acc*, precision, recall, and the F1 measure defined in Equations (3)–(6) were used here to characterize the performance of the proposed model.

(4) $$Precision\left(Prec\right)=\frac{TP}{\left(TP+FP\right)}$$

(5) $$Recall\left(Rec\right)=\frac{TP}{\left(TP+FN\right)}$$

(6) $$F1-measure\left(F1-m\right)=2\times \frac{Prec\times Rec}{\left(Prec+Rec\right)}$$

The classification results were validated based on previous studies where the KU-HAR, UCI-HAR, UniMiB SHAR, and WISDM datasets were randomly split into 70% training and 30% testing subsets. For the OPPORTUNITY dataset, the data records of the second, third, and fourth trials of participants 1, 2, and 3 were used only for testing. For PAMAP2, the testing set was also selected for the data records of subjects 5 and 6, which were never introduced to the model during training. For Sis-Fall, 10-fold cross-validation was applied.

### 4.2. Results

**Figure 4:**
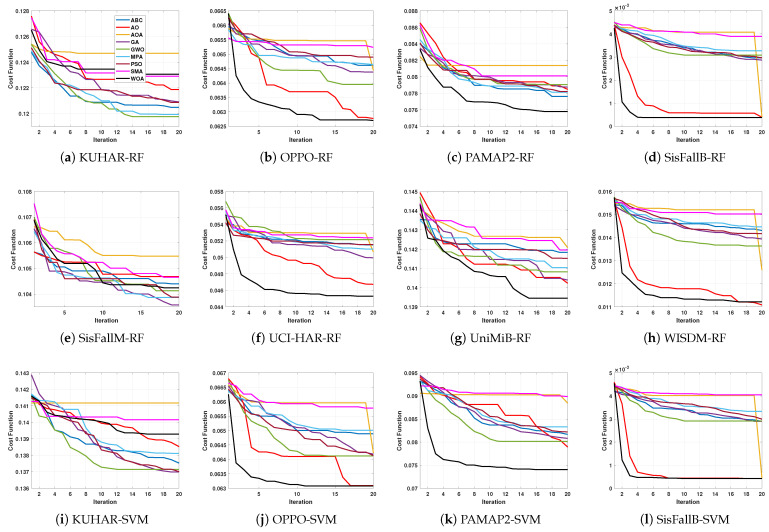

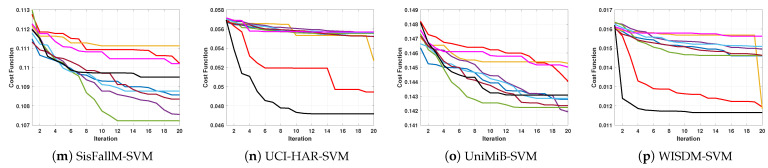
Convergence curve for applied MH optimizers for each studied dataset using RF and SVM classifiers.

In this section, the performances of all the applied MH methods are discussed and analyzed. The comparison results are given in Table 2, Table 3, Table 4, Table 5, Table 6, Table 7, Table 8, Table 9, Table 10, Table 11 and Table 12. In addition, Table 2 illustrates the average of the classification accuracy obtained using RF and SVM as classifiers to assess the different MH techniques. Additionally, the convergence curve for compared MH optimization methods using all datasets are displayed in Figure 4.

It can be seen in Table 2 that the WOA had the highest accuracy for PAMAP2, SisFallB (binary classification for fall detection), UCI-HAR, and WISDM. This was followed by the GWO, which had the best accuracy using three datasets Oppo, UniMiB SHAR, and WISDM. Meanwhile, the AO and PSO algorithms achieved the highest accuracy on two datasets, and the MPA and SMA provided better accuracy in one dataset only. In addition, we observed that RF provided better accuracy than SVM among the tested datasets and competitive algorithms.

Furthermore, Table 3 depicts the accuracy of the classification obtained using the best model (i.e., RF or SVM). Highest accuracy for KUHAR, OPPO, PAMAP2, SisFallB (binary classification), SisFallM (multi-classification), UCI-HAR, UniMiB, and WISDM was achieved with MPA, GWO, WOA, AO/WOA, AO, WOA, AO, and AO/WOA, respectively. Moreover, from these results, we can conclude that RF still performs better than SVM.

Table 4 illustrates the average reduction rate of the features obtained by each MH technique and both classifiers (i.e., RF and SVM). From these values, it can be seen that using the AO with RF and the AOA with SVM was better than other techniques on the KU-HAR dataset and the PSO with RF and the MOA with SVM were the better MH techniques on the Oppo dataset. In addition, the GWO and PSO provided better results than the other models on the PAMAP2 dataset using RF; however, by using SVM, the WOA and MPA were the best techniques. The SMA reduced the number of selected features better than the others on the SisFallB dataset using either RF or SVM. From the results of the algorithms on the SisFallM dataset, one can see that the PSO was better than the compared MH algorithms when the RF classifier was used, but by using SVM, we can see that the SMA was the best MH algorithm. For the last three datasets, we can see that the SMA had the smallest number of features on the UCI-HAR dataset with RF/SVM, UniMiB with SVM, and WISDM using SVM. However, the PSO with RF and the WOA with RF were the better algorithms according to their feature reduction rates on the UniMiB and WISDM datasets, respectively.

Table 5 lists the different classification metrics used to assess the best performing MH and classification models using the KU-HAR dataset. The AO and RF techniques showed the best performance among the other techniques on the KU-HAR dataset. As shown in Table 5, most of the activities scored more than a 90% classification score in terms of the F1 score. Activities such as Standing, Jumping, Pushing-up, and Walking-backward scored less than 86% in terms of the F1 score due to the data imbalance. Table 6 lists the results of classifying the Oppo dataset activities using the GWO and RF (SVM), where the NULL class had the majority of samples dominate the results with the highest F1 score of 96.9%. The F1 scores for the Close Drawer 1 and Close Drawer 2 activities were the lowest among all the activities and were 33.33% and 33.96%, respectively. Table 7 lists the results using the WOA and SVM on the PAMAP2 dataset. The Vacuuming activity scored lower than the other activities in F1 score and Recall. Other activity scores were close to each other due to the balanced nature of the dataset. Table 8 lists the AO and RF results on the SisFallB dataset, which is a binary classification task. The RF model showed excellent results, reaching an F1 score of 100% on both classes, Fall and No Fall. In terms of multiclass classifications of the SisFall dataset, Table 9 shows the results of SisFallM using the AO and RF on all regular activities and the Fall activity. The RF classifiers with fewer selected features using the AO compared to the original amount of features performed well on the classification of regular activities and scored 99.44% in terms of the F1 score on Fall activity. Table 10 lists the results of the WAO and RF on the UCI-HAR dataset, where the Sitting and Standing activities were less accurately classified compared to the other activities due to the similarity between the two activity signals. Table 11 lists the AO and RF results on the UniMiB SHAR dataset, where most falling activities were classified with high F1 scores. It can be seen that the Falling Rightward activity, Falling with protection, Falling backward-sitting chair, and Syncope fall had lower F1 values due to the difficult nature of the activities. Table 12 lists the WISDM dataset results using the AO and RF, which shows almost perfect scores on all classification metrics, where the RF classifier accurately differentiated between activities.

**Figure 5:**
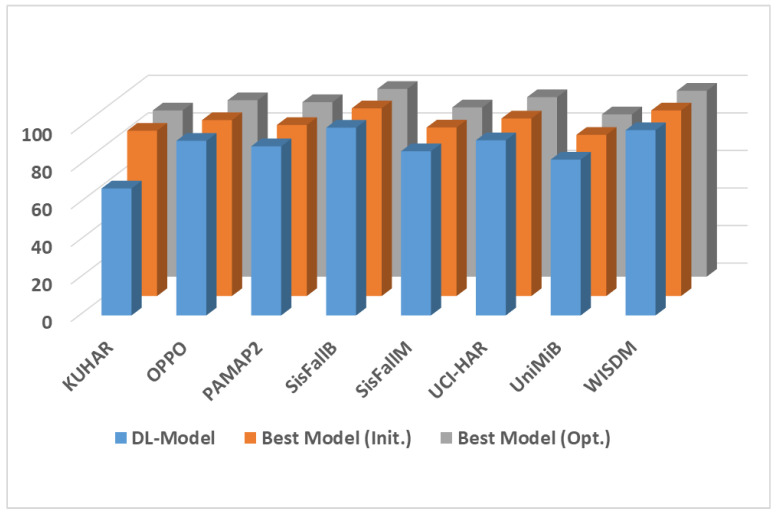
Comparison of the average accuracy of the applied DL model, DL model using RF and SVM (best model (Init.)), and the optimized DL model (best model (Opt.).

Additionally, to test the validity of the combination of MH optimization algorithms with the feature selection based on deep learning, Figure 5 presents a comparison of each applied technique (i.e., deep learning (DL) model, DL model using RF and SVM (best model (initialize results) and the optimized DL model (best model (Opt.)), which is a combination of MH and DL). The figure shows that the combination of MH and DL achieved the best results. In summary, we noticed that the applications of MH optimization algorithms showed superior performance and can be further investigated for human activity recognition and fall detection applications using body-attached sensors.

## 5. Comparison with Previous Related Studies

The accuracy reported in this simulation for the different considered datasets are compared in Table 13 to some relevant results obtained in previous studies.

From the table, we can see that the proposed approach recorded the best results in all datasets, except for PAMAP2 in which Gao et al. [45] achieved the best results, as reported in their study. The KUHAR dataset is so recent and there is not as much rich literature as the above-mentioned datasets. Binary analysis for the SisFall dataset (accuracy of SisFallB reaches 99.98%, see Table 2) revealed the excellent performance of the proposed model regarding the fall detection task, similar to the results reported by Mrozek et al. [79]. To the best of our knowledge, our results are the first multiclass analysis of the SisFall dataset.

**Table 13:** Results of related models’ accuracies (%) for common datasets

OPPO		PAMAP2		UCI-HAR		UniMiB		WISDM	
Multi-ResAtt [80]	86.85	Multi-ResAtt [80]	90.08	Daho et al. [11]	95.23	Multi-ResAtt [80]	74.94	LSTM-CNN [35]	95.01
Gao et al. [45]	82.75	Gao et al. [45]	93.16	LSTM-CNN [35]	95.31	DanHAR [45]	79.03	U-Net [81]	96.40
Teng et al. [73]	81	Teng et al. [73]	92.97	DSmT [82]	95.31	Teng et al. [73]	78.07	MHCA [83]	96.40
iSPLInception [41]	88.14	DanHAR [45]	93.16	Net-att3-pc-tanh [34]	93.83	Predsim ResNet [84]	80.33	DanHAR [45]	98.85
Proposed	93.90	Proposed	92.63	Proposed	95.51	Proposed	86.25	Proposed	98.95

## 6. Conclusions

This paper focused on applying metaheuristic (MH) optimization algorithms for daily human activity recognition and fall detection using wearable sensors such as smartphones and body-attached sensors. In general, we developed an efficient HAR and fall detection model using an integration of deep learning and MH algorithms. A new feature extraction technique based on an efficient deep learning model called ResRNN was developed to expose the related feature from the collected sensor data. Afterward, the feature selection process was implemented using nine MH algorithms, namely, the Aquila optimizer (AO), arithmetic optimization algorithm (AOA), slime mold algorithm (SMA), marine predators algorithm (MPA), genetic algorithm (GA), grey wolf optimizer (GWO), whale optimization algorithm (WOA), artificial bee colony (ABC) algorithm, and particle swarm optimization (PSO) algorithm. These MH methods were implemented to obtain optimal features, reduce computation time, and boost classification accuracy. The proposed approach was implemented in two main areas. The first was daily activity recognition (multi-classification) and the second was fall detection (binary classification). For comprehensive analysis and study, we used seven datasets that contained different and complex activities. The used datasets were UCI-HAR, Sis-Fall, WISDOM, UNIMIB-SHAR, OPPO, KU-HAR, and PAMMP2. Extensive experiments were conducted using two classifiers, support vector machine (SVM) and random forest (RF). The findings of this study showed that the MH optimization algorithms showed significant performance in HAR and fall detection applications. This study opens the possibilities of the MH to be applied for sensing applications. We suggest using more advanced methods, such as modified MH optimization methods integrated with intelligent search mechanisms, that may provide more robust results, such as levy flight, opposition-based learning, and a hybridization of two MH algorithms.

## Figures and Tables

**Table 1:** Parameter settings of used MH algorithms.

Algorithm	Parameters
ABC	a=1
AO	α=0.1, δ=0.1, u=0.0265, $r_{0}=10$, ω=0.005, $\phi_{0}=3\pi /2$,
AOA	$MOP_{Max}=1$, $MOP_{Min}=0.2$, α=2.5, μ=0.1
GA	Crossover =40%, Mutation =15%
GWO	a:2→0
MPA	FADs=0.2, P=0.5
PSO	Inertia weight (*w*) = 1, $w_{damp}=0.99$, $c_{1}=2$, $c_{2}=2$
SMA	z=0.03
WOA	a:2→0, a2: −1→−2, b=1

**Table 2:** Average classification accuracy (%) of applied MH optimizers

	KUHAR		OPPO		PAMAP2		SisFallB		SisFallM		UCI-HAR		UniMiB		WISDM	
	RF	SVM	RF	SVM	RF	SVM	RF	SVM	RF	SVM	RF	SVM	RF	SVM	RF	SVM
ABC	88.03	86.61	93.73	93.84	92.06	92.13	99.97	99.97	89.59	89.50	94.85	94.76	85.74	86.02	98.83	98.83
AO	88.3	86.83	93.81	93.76	92.44	92.2	99.98	99.97	89.92	89.33	95.37	95.07	86.17	85.75	98.95	98.87
AOA	87.89	86.66	93.64	93.61	92.23	91.44	99.97	99.97	89.82	89.26	95.12	94.9	86	85.69	98.79	98.85
GA	87.95	86.7	93.74	93.89	91.67	92.25	99.97	99.97	89.47	89.62	95.15	94.75	85.87	86.1	98.85	98.82
GWO	88.4	86.69	93.90	93.9	92.5	92.31	99.97	99.97	89.93	89.65	95.13	94.77	86.25	86.1	98.95	98.83
MPA	88.41	86.6	93.88	93.86	92.58	92.02	99.97	99.97	89.96	89.50	95.27	94.75	86.22	86.02	98.94	98.82
PSO	88.32	86.71	93.87	93.91	92.58	92.1	99.97	99.97	90.00	89.54	95.21	94.78	86.21	86.05	98.95	98.82
SMA	87.77	86.38	93.72	93.76	91.63	91.34	99.97	99.97	89.52	89.37	94.62	94.82	85.71	85.82	98.85	98.8
WOA	88.31	86.7	93.81	93.75	92.57	92.63	99.98	99.97	89.91	89.33	95.51	95.28	86.14	85.85	98.95	98.87

**Table 3:** Classification accuracy (%) of the best model for each dataset

	KUHAR	OPPO	PAMAP2	SisFallB	SisFallM	UCI-HAR	UniMiB	WISDM
	RF	RF/SVM	SVM	RF	RF	RF	RF	RF
ABC	88.37	93.79	92.36	99.97	89.92	95.11	85.96	98.85
AO	88.53	93.92	92.48	100	90.13	95.49	86.44	98.99
AOA	88.09	93.73	91.57	99.97	89.86	95.22	86.13	98.81
GA	88.07	93.78	92.38	99.97	89.59	95.39	86.3	98.91
GWO	88.49	93.95	92.42	99.97	89.98	95.42	86.33	98.97
MPA	88.52	93.91	92.3	99.97	90.04	95.39	86.35	98.97
PSO	88.44	93.91	92.32	99.97	90.1	95.25	86.33	98.97
SMA	88.06	93.86	91.71	99.97	89.68	94.74	86.04	98.87
WOA	88.42	93.83	92.95	100	89.98	95.59	86.18	98.99

**Table 4:** Average percentage of feature reduction (%) of applied MH optimizers

	KUHAR		OPPO		PAMAP2		SisFallB		SisFallM		UCI-HAR		UniMiB		WISDM	
	RF	SVM	RF	SVM	RF	SVM	RF	SVM	RF	SVM	RF	SVM	RF	SVM	RF	SVM
ABC	51.41 ± 4.6	50 ± 6.04	60.31 ± 5.07	60.94 ± 3.87	57.66 ± 4.44	61.72 ± 3.39	73.13 ± 2.51	73.75 ± 2.97	51.56 ± 3.67	53.13 ± 8.15	56.72 ± 3.58	66.09 ± 4.04	54.22 ± 7.5	55.78 ± 7.83	63.91 ± 5.63	69.22 ± 2.07
AO	39.69 ± 14.7	17.97 ± 14.53	85.47 ± 8.96	87.19 ± 4.93	63.13 ± 13.33	82.97 ± 8.44	98.44 ± 1.22	98.75 ± 0.55	51.09 ± 3.91	53.91 ± 12.59	90.78 ± 5.02	93.28 ± 2.07	66.41 ± 16.4	69.84 ± 13.85	92.81 ± 2.95	92.5 ± 3.44
AOA	51.88 ± 8.44	8.59 ± 24.6	85.31 ± 10.71	90.47 ± 2.95	55.31 ± 2.59	62.34 ± 24.16	98.91 ± 0.55	98.44 ± 1.41	52.66 ± 3.21	51.88 ± 5.41	76.25 ± 27.52	78.13 ± 28.52	64.69 ± 13.33	63.44 ± 12.95	93.44 ± 4.1	95.31 ± 1.87
GA	48.75 ± 5.13	46.56 ± 4.45	59.53 ± 2.39	62.66 ± 3.83	54.06 ± 4.97	58.59 ± 4.47	74.06 ± 2.05	73.75 ± 4.28	56.25 ± 3.61	51.41 ± 4.32	55.63 ± 8.7	62.66 ± 5.54	53.44 ± 4.72	56.72 ± 4.51	65.63 ± 3.94	67.34 ± 2.39
GWO	50.47 ± 3.78	46.25 ± 2.59	64.53 ± 4.77	62.34 ± 3.42	51.88 ± 6.91	59.69 ± 2.3	71.88 ± 3.46	73.59 ± 4.27	55.47 ± 4.9	51.72 ± 4.76	59.84 ± 7.13	61.72 ± 4.06	52.5 ± 2.17	53.13 ± 4.47	67.5 ± 2.19	69.06 ± 6.35
MPA	47.81 ± 1.3	45.31 ± 4.3	59.38 ± 4.64	57.5 ± 5.64	53.28 ± 11.23	56.56 ± 2.41	70 ± 5.37	69.38 ± 2.17	55.16 ± 2.79	51.72 ± 5.76	57.81 ± 3.24	62.97 ± 3.21	53.13 ± 1.87	54.69 ± 4.53	60.31 ± 3.7	65.47 ± 1.64
PSO	46.88 ± 3.67	44.69 ± 6.61	57.5 ± 8.44	61.09 ± 5.63	51.88 ± 2.19	59.84 ± 4.83	73.13 ± 4.28	72.81 ± 1.64	50.78 ± 11.11	51.72 ± 5.93	58.28 ± 6.73	64.22 ± 4.15	50 ± 5.96	56.88 ± 3.11	61.72 ± 2.74	70.16 ± 5.59
SMA	46.88 ± 2.74	46.09 ± 5.52	58.59 ± 4.3	59.69 ± 3.44	55.31 ± 5.5	57.97 ± 6.3	63.75 ± 2.51	62.19 ± 1.52	53.75 ± 3.27	50.47 ± 5.59	53.59 ± 4.34	56.09 ± 10.47	55.78 ± 5.32	53.13 ± 5.79	60.62 ± 2.7	62.5 ± 3
WOA	47.81 ± 1.3	45.31 ± 4.3	59.38 ± 4.64	57.5 ± 5.64	53.28 ± 11.23	56.56 ± 2.41	70 ± 5.37	69.38 ± 2.17	55.16 ± 2.79	61.09 ± 11.43	57.81 ± 3.24	62.97 ± 3.21	53.13 ± 1.87	54.69 ± 4.53	60.31 ± 3.7	65.47 ± 1.64

**Table 5:** Classification rates (%) of the best model of the **KUHAR** dataset using RF.

Activity	Pre	Rec	F1
Standing	76.29	73.32	74.77
Sitting	98.3	95.7	96.98
Talking-Sit	94.8	96.23	95.51
Talking-Stand	92.86	95.79	94.3
Standing-Sit	96	92.31	94.12
Laying	93.85	94.38	94.12
Laying-Stand	95.2	91.21	93.16
Picking	95.71	95.3	95.5
Jumping	53.36	91.81	67.5
Pushing-up	89.82	81.82	85.63
Sitting-up	97.11	95.89	96.5
Walking	98.48	99.23	98.86
Walking-backward	80.62	33.64	47.47
Walking-circle	97.19	98.11	97.65
Running	96.52	97	96.76
Stairs up	96.94	95	95.96
Stairs down	97.84	94.44	96.11

**Table 6:** Classification rates (%) of the best model of the **OPPO** dataset using either RF or SVM.

Activity	Pre	Rec	F1
NULL	95.37	98.49	96.9
Open Door 1	94.03	80.77	86.9
Open Door 2	97.5	90.7	93.98
Close Door 1	91.23	81.25	85.95
Close Door 2	96.67	93.55	95.08
Open Fridge	89.04	83.87	86.38
Close Fridge	88.99	71.85	79.51
Open Dishwasher	79.63	69.35	74.14
Close Dishwasher	68.52	66.07	67.27
Open Drawer 1	46.88	62.5	53.57
Close Drawer 1	46.67	25.93	33.33
Open Drawer 2	57.14	64	60.38
Close Drawer 2	27.27	45	33.96
Open Drawer 3	68.85	79.25	73.68
Close Drawer 3	97.14	70.83	81.93
Clean Table	81.4	57.38	67.31
Drink from Cup	91.94	58.76	71.7
Toggle Switch	87.5	60.87	71.79

**Table 7:** Classification rates (%) of the best model of the **PAMAP2** dataset using SVM.

Activity	Pre	Rec	F1
Lying	95.77	93.61	94.68
Sitting	97	95.67	96.33
Standing	84.06	91.34	87.55
Normal Walking	86.19	96.98	91.27
Running	99.78	98.7	99.24
Cycling	96.43	99.61	97.99
Nordic Walking	95.71	95.71	95.71
Ascending Stairs	91.21	95.82	93.46
Descending Stairs	95.2	93.83	94.51
Vacuuming	98.58	61.5	75.75
Ironing	83.99	90.79	87.26
Rope Jumping	97.15	99.09	98.11

**Table 8:** Classification rates (%) of the best model of the **SisFallB** dataset using RF.

Activity	Pre	Rec	F1
Fall	100	100	100
No Fall	100	100	100

**Table 9:** Classification rates (%) of the best model of the **SisFallM** dataset using RF.

Activity	Pre	Rec	F1
Walking Slowly	98.71	98.71	98.71
Walking Quickly	100	100	100
Jogging Slowly	98	98.39	98.2
Jogging Quickly	98.64	97.75	98.19
Walking upstairs and downstairs slowly	92.25	92.58	92.42
Walking upstairs and downstairs quickly	77.78	81.29	79.5
Sitting in a half-height chair, waiting a moment, and getting up slowly	79.86	77.08	78.45
Sitting in a half-height chair, waiting a moment, and getting up quickly	76.28	81.51	78.81
Sitting in a low-height chair, waiting a moment, and getting up slowly	70	74.34	72.1
Sitting in a low-height chair, waiting a moment, and getting up quickly	80	67.8	73.39
Sitting a moment, trying to get up, and collapsing into a chair	86.55	80.47	83.4
Sitting a moment, lying slowly, waiting a moment, and sitting again	93.91	93.91	93.91
Sitting a moment, lying quickly, waiting a moment, and sitting again	88.46	84.15	86.25
Being on one’s back, changing to lateral position, waiting a moment, and changing to one’s back	95.97	95.97	95.97
Standing, slowly bending at knees, and getting up	86.15	78.87	82.35
Standing, slowly bending without bending knees, and getting up	87.07	88.28	87.67
Standing, getting into a car, remaining seated, and getting out of the car	82.49	91.76	86.88
Stumbling while walking	97.62	95.35	96.47
Gently jumping without falling (trying to reach a high object)	88.51	82.8	85.56
Falling	99.44	99.44	99.44

**Table 10:** Classification rates (%) of the best model of the **UCI-HAR** dataset using RF.

Activity	Pre	Rec	F1
Walking	99.59	97.98	98.78
Walking Upstairs	98.31	98.51	98.41
Walking Downstairs	96.74	99.05	97.88
Sitting	92.05	84.93	88.35
Standing	88.43	93.42	90.86
Laying Down	99.08	100	99.54

**Table 11:** Classification rates (%) of the best model of the **UniMiB-SHAR** dataset using RF.

Activity	Pre	Rec	F1
Standing Up From Sitting	64.33	68.24	66.23
Standing Up From Lying	87.5	77.21	82.03
Walking	76.07	75.61	75.84
Running	64.33	65.58	64.95
Going UpS	77.7	70.59	73.97
Jumping	70.06	72.37	71.2
Going DownS	94.41	96.43	95.41
Lying DownFS	92.06	88.55	90.27
Sitting Down	92.75	88.61	90.63
Falling Forward	98.14	98.6	98.37
Falling Rightward	69.79	73.63	71.66
Falling Backward	99.14	99.31	99.22
Hitting Obstacle	73.68	73.68	73.68
Falling With Protection	72.37	77.46	74.83
Falling Backward-Sitting Chair	81.82	60	69.23
Syncope Fall	52.17	61.15	56.3
Falling Leftward	98.28	97.72	98

**Table 12:** Classification rates (%) of the best model of the **WISDM** dataset using RF.

Activity	Pre	Rec	F1
Walking	97.09	96.01	96.54
Walking Upstairs	100	98.81	99.4
Walking Downstairs	98.46	100	99.22
Sitting	95.83	95.67	95.75
Standing	100	99.95	99.97
Jogging	99.38	99.75	99.57

## Data Availability

The data are publicly available as described in the main text.
